# Study on Visual Detection Algorithm of Sea Surface Targets Based on Improved YOLOv3

**DOI:** 10.3390/s20247263

**Published:** 2020-12-18

**Authors:** Tao Liu, Bo Pang, Shangmao Ai, Xiaoqiang Sun

**Affiliations:** 1 College of Intelligent Systems Science and Engineering, Harbin Engineering University, Harbin 150001, China; liutao@hrbeu.edu.cn (T.L.); pangbo@hrbeu.edu.cn (B.P.); sunxiaoqiang@hrbeu.edu.cn (X.S.); 2 College of Shipbuilding Engineering, Harbin Engineering University, Harbin 150001, China

**Keywords:** YOLO v3, anchor-setting, target detection, feature fusion, connection of cross-feature maps, buoys, ships

## Abstract

Countries around the world have paid increasing attention to the issue of marine security, and sea target detection is a key task to ensure marine safety. Therefore, it is of great significance to propose an efficient and accurate sea-surface target detection algorithm. The anchor-setting method of the traditional YOLO v3 only uses the degree of overlap between the anchor and the ground-truth box as the standard. As a result, the information of some feature maps cannot be used, and the required accuracy of target detection is hard to achieve in a complex sea environment. Therefore, two new anchor-setting methods for the visual detection of sea targets were proposed in this paper: the average method and the select-all method. In addition, cross PANet, a feature fusion structure for cross-feature maps was developed and was used to obtain a better baseline cross YOLO v3, where different anchor-setting methods were combined with a focal loss for experimental comparison in the datasets of sea buoys and existing sea ships, SeaBuoys and SeaShips, respectively. The results showed that the method proposed in this paper could significantly improve the accuracy of YOLO v3 in detecting sea-surface targets, and the highest value of mAP in the two datasets is 98.37% and 90.58%, respectively.

## 1. Introduction

As human maritime activities have gradually expanded with the rapidly growing marine economy, countries have paid increasing attention to the protection of coastlines. Compared with other perception technologies, the light vision-based perception technology is easier to effectively identify the water surface target since the optical image contains more detailed information about the target area. Studying the sea-surface target detection technology based on light vision can help the unmanned surface vehicle (USV) to extract information of coasts of a large area, sailing vessels, and floating obstacles (such as floating bodies and floating debris) in its operation process, and helps to achieve autonomous avoidance of obstacles, thereby avoiding shipwreck or collision between vessels and improving their own survival and operational capabilities. Therefore, sea-surface target detection has become a research focus in the field of marine security and is closely related to national defense security, maritime surveillance, and maritime rescue. Visual image target detection is a key technology to improve the intelligence of sea-surface unmanned systems. Traditional target detection algorithms consist of feature extractors and classifiers. The commonly used feature extractors include scale-invariant feature transform (SIFT), histogram of oriented gradient (HOG), etc., and classifiers include support vector machine (SVM). In recent years, target detection algorithms based on neural networks have developed fast thanks to the improvement of the operational capability of computers. Convolutional neural network (CNN), as the backbone network of modern target detection algorithms, possesses a strong ability to extract features from visible images and has achieved rapid development since AlexNet [1] has made tremendous progress in the realm of image classification. In the InceptionNet series, the branch structure was proposed to reduce the number of parameters of the neural networks [2,3,4]. The residual structure presented by He et al. allows for deeper CNN [5]. Xie et al. introduced the cardinality of a new dimension into the neural network structure [6], which improved the efficiency and accuracy of the neural network. DenseNet and CSPNet can reuse shallow features, which is conducive to gradient spreading [7,8]. In addition to selecting a good backbone network, multiscale feature map fusion is also a key component of modern target detection algorithms. He et al. proposed a feature pyramid structure to conduct top-down feature fusion [9]. Based on this, Liu et al. proposed a bottom-up path [10]. BiFPN and AFSS [11,12] introduced the concept of weight on the basis of [9] so as to assign different weights to different feature maps before feature fusion and allow the network to learn the weight coefficients autonomously.

The target detection algorithms based on neural networks can be divided into one-stage algorithms and two-stage algorithms. Representatives of the two-stage algorithms include RCNN, fast RCNN, faster RCNN, etc. [13,14,15]. In [13], the neural network was applied to target detection for the first time, and the traditional feature extractor was replaced with CNN. In [15], the concepts of region proposal network (RPN) and anchor were proposed, and the speed and accuracy were significantly improved. One-stage algorithms are represented by YOLO v1, YOLO v2, YOLO v3, YOLO v4, SSD, etc. [16,17,18,19,20]. Compared with the two-stage algorithms, one-stage algorithms can directly output the category and bounding box coordinates of the detected object, with the advantage in speed. At present, a number of target detection algorithms based on deep learning have been successfully applied in sea-surface target detection. The attention mechanism is often introduced into the target detection algorithm to improve the accuracy of target detection, but it will reduce the detection speed of the network [21,22]. Based upon faster RCNN, Xiao et al. proposed to increase the number of target samples by reducing the batch size, and relabeled the dataset with only one category according to the features of the ships in the SAR image, and gave different labels to different ships [23]. Zhang et al. compared the detection results of faster RCNN and mask RCNN in detail in the dataset of remote sensing map images [24]. Liu et al. put forth a rotated region-based CNN to detect ships facing different sea surfaces [25]. Dong et al. developed a multi-angle box-based rotation insensitive object detection structure to overcome the learning difficulties of the detector caused by the uncertain direction angle of ship targets in the dataset of remote sensing images [26]. The more complex feature fusion structure will improve accuracy, but it will also reduce detection speed and increase network complexity [27,28]. Based on YOLO v3, Li et al. introduced the DenseNet structure to enhance the ability of YOLO v3 to extract features and conducted an experiment in the dataset of unmanned sea-surface vehicles [29]. Dai et al. presented a two-stage detector to detect small targets in SAR images. The network is composed of three subnets: the fusion feature extractor network (FFEN), region proposal network (RPN), and refine detection network (RDN) [30]. Chen et al. combined a generative adversarial network (GAN) with a target detector to improve the ability to detect small target ships [31]. Su et al. studied RetinaNet-Plus on the basis of RetinaNet and applied it to the task of ship detection from SAR images [32].

However, compared to the SAR image dataset and the high-resolution ship images on the Internet, the actual sea-surface target imaging is often affected by a water spray, changes of light, and sway of boat and becomes unclear, which will reduce the accuracy of detection. In order to solve the sea-surface target detection problem, the pursuit of higher accuracy often brings additional calculations, such as larger backbone networks and feature fusion structures, and additional components such as attention mechanisms. Balancing the speed and accuracy of the target detection network is the major difficulty of the sea-surface target detection task. The anchor-setting method proposed in this paper can improve the accuracy of target detection without reducing the detection speed, which meets the needs of sea target detection tasks. YOLO v3 has a simple structure, a small network size, and does not add too many components, which can be more focused on the optimization scheme proposed in this paper. YOLO v3 can balance speed and accuracy well, has strong stability, and is suitable for various practical tasks.

In this paper, for the unmanned surface vehicle, improved YOLO v3 was proposed and applied to the target detection of the visible images of the sea surface. The main contribution of this paper is manifested in the following three aspects.

First, the setting method for the anchor of YOLO v3 was improved. In this paper, two anchor-setting methods that do not require a manual setting of hyperparameters were proposed, namely, the average method and the select-all method. In the previous anchor-setting methods, the degree of overlap between the anchor and the ground-truth box was often regarded as the only indicator, while the information of some feature maps was ignored. The two proposed new methods in this paper not only take the degree of overlap between the anchor and the ground-truth box into consideration but also consider how to comprehensively use the information of each feature map.

Second, the feature fusion structure in YOLO v3 was improved. Feature fusion structure, which can combine the location information of the low-level feature maps with the semantic information of the high-level feature maps, has become an indispensable component of the target detection network. The feature fusion structure in YOLO v3 is the feature pyramid network (FPN) that only considers the downward spreading of high-level semantic information. By contrast, the path aggregation network (PANet) has two different fusion paths so that the information of different feature maps can be fused in a more comprehensive way. In this paper, the PANet-based cross PANet feature fusion structure was proposed, which can make full use of the strongest positioning information and semantic information in the feature maps.

Third, in this paper, the dataset of SeaBuoys, SeaBuoys, was created based on the actual sea-surface conditions, and a large number of comparative experiments were conducted in SeaBuoys and the existing dataset of SeaShips, SeaShips [33]. The final experimental results demonstrated that these improvements enhanced the ability of YOLO v3 to detect sea-surface targets.

## 2. Methods

This section describes in detail different anchor-setting methods, the methods of improving the feature fusion structure and the improvement of the loss function, and finally, a better YOLO v3 baseline was obtained.

### 2.1. Threshold Method

The anchor-based target detection algorithm needs to assign a binary category label to each anchor. The threshold method is widely applied in the YOLO series of algorithms and has three steps to set positive anchors. First, a threshold x (0 < x < 1) is set for the network; second, the intersection-over-union (IOU) between all anchors and ground-truth boxes is computed; third, the anchors larger than the threshold x are set as positive anchors. After these three steps, some targets still do not have qualified positive anchors to match. In this case, the anchor whose IOU with the ground-truth box is the largest can be selected as the positive anchor, of which the confidence label is set to 1, and the confidence loss, category loss and bounding box coordinates loss need to be calculated. The smaller the x, the greater the number of positive anchors. Too many positive anchors will make network training difficult, and too few will increase the imbalance between the number of positive anchors and negative anchors. The threshold x is usually selected manually, and the value of x in this paper is 0.3.

In terms of the setting of negative anchors, a threshold y needs to be set during the training process. All anchors whose IOU with the ground-truth box greater than the threshold y do not participate in any loss calculation, and those with IOU smaller than y are set as negative anchors, of which the confidence label is set to 0, and only the confidence loss is computed. The threshold y is usually selected manually. The smaller the value of y, the smaller the number of negative anchors. YOLO v3 has the problem of imbalance between the number of positive anchors and negative anchors, so y should be a small value. In this paper, the y value takes 0.3. Algorithm 1 shows the process of setting positive anchors by the threshold method.
**Algorithm 1.** The process of setting positive anchors by the threshold method.Suppose the list of multiscale feature maps is
$F=\left[F_{1},F_{2}\cdots F_{K}\right]$
Step 1: Set a threshold *x* (0 < *x* < 1) and *K* = 1Step 2: Select $F_{K}$Step 3: Find the grid where the center of the target in $F_{K}$ is locatedStep 4: Calculate the $IOU=\left(a_{1},a_{2}\cdots a_{n}\right)$ between all anchors and  ground-truth boxes in the selected gridStep 5: Select the anchor corresponding to $a_{i}\left(a_{i}>x\right)$ and set it to positive anchorStep 6: Update *K* to *K* + 1, if *K* > len(F), end the process, or return to Step 3

### 2.2. Average Method

The threshold method selects the anchors with shape and size close to those of the ground-truth boxes as the positive anchors, which is a reasonable idea. However, the positive anchors selected by the threshold method may be concentrated in a certain feature map, and some feature maps have no chance to predict the coordinates of the ground-truth box. As a result, the information on some feature maps will be ignored. Therefore, it is necessary to ensure that not only the anchors with shape and size close to those of the ground-truth boxes are set as positive anchors, but also each feature map has the opportunity to predict the ground-truth box.

The average method proposed in this paper removes the threshold limit when matching positive anchors to the target. The average method consists of 3 steps. First, a feature map L that outputs the final result is determined. The second step is to find grid A where the target center in L is located. Third, the anchor with the largest IOU with the ground-truth box in A is computed and selected and set as a positive anchor. YOLO v3 has 3 different feature maps to output the final result; therefore, the average method will match 3 positive anchors to each target, with one for each feature map. The method of setting a negative anchor is the same as the threshold method. The average method does not need manual selection of the threshold, and the selection of the threshold often has a great impact on the detection result. Algorithm 2 shows the process of setting the positive anchor by the average method.
**Algorithm 2.** The process of setting positive anchors by the average methodSuppose the list of multiscale feature maps is $F=\left[F_{1},F_{2}\cdots F_{K}\right]$
Step 1: Select $F_{K}$ and *K* = 1Step 2: Find the grid where the center of the target $F_{K}$ is locatedStep 3: Calculate the $IOU=\left(a_{1},a_{2}\cdots a_{n}\right)$ between all anchors and    ground-truth boxes in the selected gridStep 4: Select the anchor corresponding to $MAX(a_{i})$ and set it to positive anchorStep 5: Update *K* to *K* + 1, if *K* > len(*F*), end the process, or return to Step 2

### 2.3. Select-All Method

In order to compare with the average method, the select-all method was also proposed in this paper. Compared to the average method, the select-all method can increase the number of positive anchors, but it also brings greater difficulty to network training and reduces the fault tolerance for network prediction.

The select-all method also removes the threshold limit, and there are 3 steps in total. The first step is to determine a feature map M that outputs the final result. Second, the grid B, where the target center in M is located, is found. The third step is to set all anchors in B as positive anchors. YOLO v3 has 3 different feature maps to output the final result. Three anchors will be preset in each grid, which means that the select-all method will match 9 positive anchors to each target, with 3 in each feature map. The method of selecting negative anchors is the same as the threshold method. Algorithm 3 exhibits the process of setting a positive anchor by the select-all method.
**Algorithm 3.** The process of setting positive anchors by the select-all method.Suppose the list of multiscale feature maps is $F=\left[F_{1},F_{2}\cdots F_{K}\right]$
Step 1: Select $F_{K}$ and *K* = 1Step 2: Find the grid where the center of the target in $F_{K}$ is locatedStep 3: Select all anchors in the grid and set them to positive anchorsStep 4: Update *K* to *K* + 1, if *K* > len(*F*), end the process, or return to Step 2

### 2.4. Cross PANet

The feature fusion structure in YOLO v3 is FPN, which fuses the information of different feature maps from top to bottom. Although PANet uses the same feature pyramid structure as FPN, the difference lies in that PANet adds a bottom-up feature fusion path on the basis of FPN, and the strong positioning information in the lower layer is spread upward, which is equivalent to two feature fusions in different directions, allowing each feature map predicting the final result to have better semantic information and location information.

**Figure 1:**
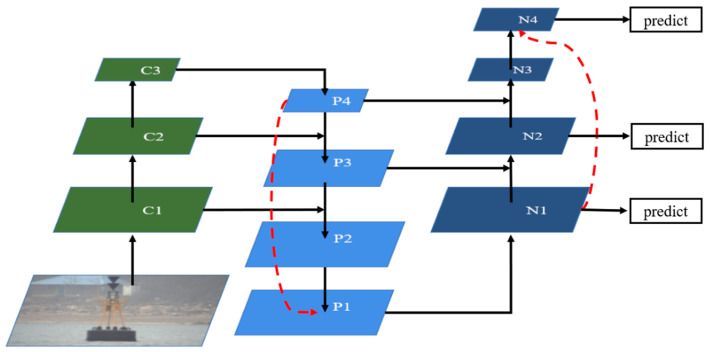
Structure of cross path aggregation network (PANet).

The way information is disseminated in the neural network is crucial. The most common pattern is that the output of the last neural network is the input of the next neural network. However, in the pyramid structure, the information of two non-adjacent feature maps cannot be directly fused. In order to allow the information to be better disseminated during feature fusion, a feature fusion structure for cross-feature maps was proposed based upon PANet. In the top-down path, the smallest feature map is upsampled twice and fused with the largest feature map, while in the bottom-up path, the largest feature map is downsampled twice and fused with the smallest feature map. Such structure is called cross PANet, where the direct fusion between the smallest and largest feature maps can more directly make use of the strongest semantic information and location information in the multiscale feature maps. There are 3 multiscale feature maps in YOLO v3 to predict the final result. Concat was used for the fusion between different feature maps in cross PANet. Figure 1 shows the structure of cross PANet.

In Figure 1, C1, C2, C3 are the feature maps of different sizes output by the backbone network of YOLO v3, and the red connecting line represents the fusion process of cross-feature maps. In the top-down path, P4 is upsampled twice and fused with P2 to generate P1. Among P2, P3, and P4, P4 has the strongest semantic information, and P2 has the strongest location information. In the bottom-up path, N1 is downsampled twice and fused with N3 to generate N4 so that the strongest semantic information and the strongest location information are also integrated. Finally, N1, N2, N4 predict the category and the coordinates of the bounding box of the target.

### 2.5. Loss Function

The loss function of the target detection network generally consists of three parts, namely the confidence loss, the loss of bounding box coordinates and the category loss. The commonly used loss function of bounding box coordinates is the mean square error (MSE) loss function based on the Euclidean distance, and IOU can better express the overlap between two bounding boxes compared with Euclidean distance. However, when there is no overlap between two bounding boxes, IOU is 0, (which makes the network unable to use the gradient to adjust parameters. Therefore, Generalized Intersection over Union (GIOU) loss [34] was used as the loss function of bounding box coordinates of YOLO v3. Compared to MSE, GIOU loss can better express the degree of overlap between two different bounding boxes, which can satisfy the actual needs of target detection. Meanwhile, the value of GIOU also changes when the two bounding boxes do not overlap. GIOU and GIOU loss are defined as follows:

(1) $$GIoU=IoU-\frac{\left|C-(A\cup B)\right|}{C}$$

(2) GIoUloss=1−GIoU

In Formula (1), A is the ground-truth box, B is the predicted bounding box, and C is the smallest rectangular box that completely encloses A and B.

The focal loss was introduced into the confidence loss function. During the training, focal loss assigns larger weights to samples that are difficult to classify, which is a key method to solve the problem of sample imbalance. The confidence loss function after the focal loss is introduced and defined as follows:

(3) $${Loss}_{conf}=-\alpha_{1}{\left(y_{GT}-y_{p}\right)}^{\gamma }\times y_{GT}\log y_{p}-\alpha_{2}{\left(y_{GT}-y_{p}\right)}^{\gamma }\times \left(1-y_{GT}\right)\log\left(1-y_{p}\right)$$

where $y_{GT}$ is the ground-truth of the confidence, the foreground is 1, and the background is 0, $y_{p}$ which is the network output value of the confidence $\alpha_{1}=\alpha_{2}=1$,γ=2.

The binary cross-entropy loss function was still adopted for the classification loss function, which is defined as follows:

(4) $${Loss}_{cls}=-C_{GT}\log C_{P}-\left(1-C_{GT}\right)\log\left(1-C_{p}\right)$$

where $C_{GT}$ is the ground-truth of the target category, $C_{P}$ is the network output value of the target category.

The complete loss function is composed of three parts: loss of bounding box coordinates, confidence loss and category loss, as shown in Formula (5), where *i* represents the sequence number of the feature map that finally outputs the predicted value.

(5) $${Loss}_{total}=\sum_{i=1}^{3}GIoUloss^{i}+Loss_{conf}^{i}+Loss_{cls}^{i}$$

Cross PANet, GIOU loss and threshold method were employed in this study to infer a better baseline cross YOLO v3, where the threshold method, averaged method, select-all method and focal loss can be combined in different ways and tested in different datasets of sea-surface targets.

## 3. Dataset of Sea Surface Targets

### 3.1. Dataset of Sea Surface Buoys

**Figure 2:**
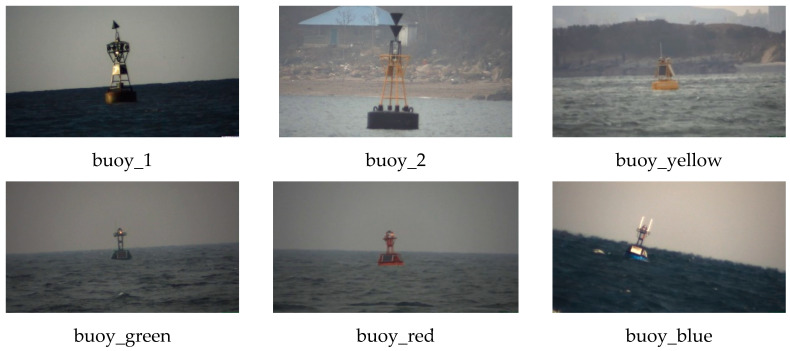
The appearance and corresponding labels of different buoys.

A dataset of sea-surface buoys was created based on the actual situation of the sea surface and named SeaBuoys. All the images were collected from the real sea surface by a simulated camera. SeaBuoys consists of 5751 images in RGB format, including six different categories of buoys, all of which are of the same size 1024×576. The largest difference in the proportion of each category is less than 3%. Table 1 shows the specific information of SeaBuoys, and Figure 2 displays the appearance of 6 types of buoys in SeaBuoys.

### 3.2. Dataset of Sea Surface Ships

**Figure 3:**
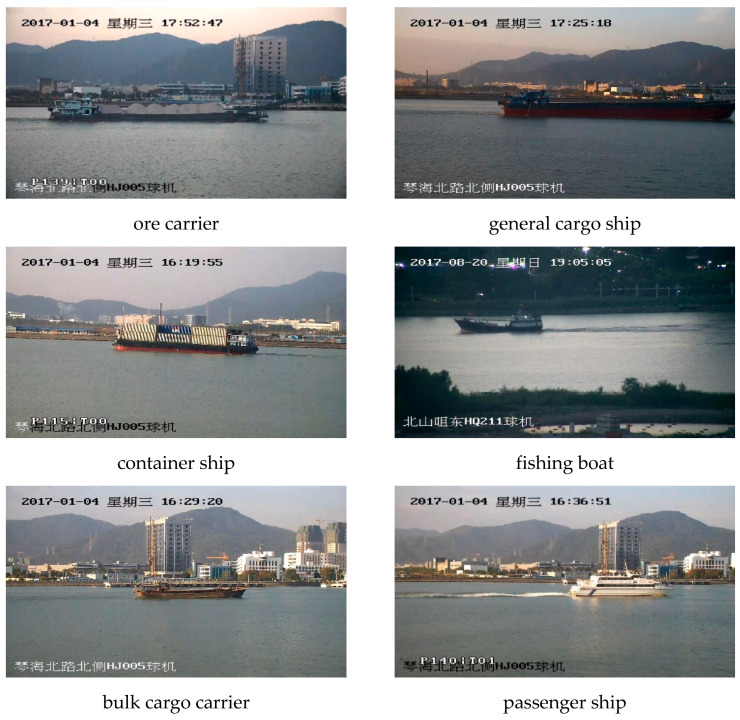
The appearance and corresponding labels of different ships in the SeaShips dataset.

Experiments were also conducted in SeaShips, which includes 31,455 images with a size of 1920×1080 in RGB format containing 6 types of ship targets (7000 publicly available)(ore carrier, bulk cargo carrier, general cargo ship, container ship, fishing boat, and passenger ship). Figure 3 shows the appearance of different ships in SeaShips.

## 4. Results

### 4.1. Experimental Details

In order to ensure the fairness of the experiment, all experiments in this paper were conducted on the same hardware platform and under the same software framework. The graphics processing unit (GPU) adopts a single NVIDIA GeForce RTX 2080TI (NVIDIA, Santa Clara, California, USA), and the central processing unit (CPU) is i7-9700 (Intel, Santa Clara, California, USA). The running memory is 32 G, and the software framework is the TensorFlow 2.1.0-GPU version (Google, Santa Clara, California, USA).

**Figure 4:**
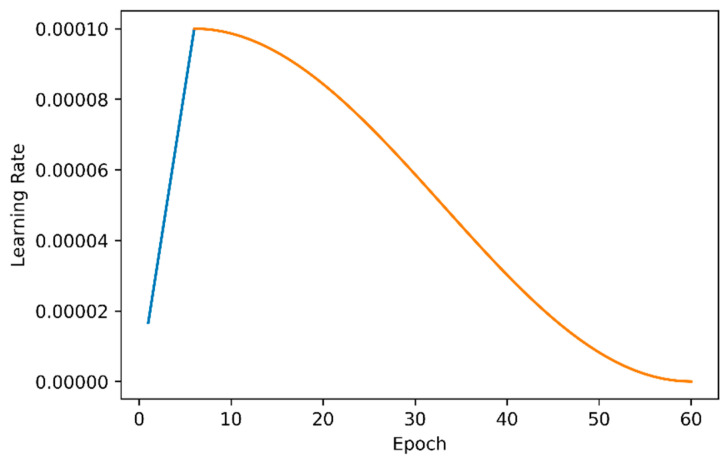
Variation curve of the learning rate, the blue part is the warm-up process, and the orange part is the decreasing process at the cosine learning rate.

In terms of network training, all experiments were optimized by gradient descent method, with the weight decay coefficient of 0.0005 and the impulse of 0.9. Regarding the data enhancement strategy, horizontal flip and scaling were used. The size of the input image during training is 416×416, and the size of the input image during the test is 544×544. The variation of learning rate was realized by two methods: cosine learning rate and warm-up [35]. The number of rounds of the warm-up is set to 6. In the first 6 rounds of training, the learning rate was increased by a small value to the initial learning rate of 0.0001 and then decreased to the final learning rate of 0.000001 according to the law of cosine learning rate. A total of 60 rounds of training were performed. The downward trend of the cosine function meets the requirements of the neural network for the variation of the learning rate. Figure 4 shows the variation trend of the learning rate. For the experimental results, we will analyze multiple indicators of mean average precision (mAP), inference time, ground truth (GT), true positive (TP) and false positive (FP).

### 4.2. Ablation Experiment in SeaBuoys

In SeaBuoys, 80% of the images were classified into a training set, and 20% were classified into a test set. First, an experiment was conducted on cross YOLO v3 in SeaBuoys. Second, the effect of the threshold method, the average method and the select-all method was verified by combining them with focal loss in different ways, with cross YOLO v3 as the baseline. The experimental results are shown in the table below.

In Table 2, the mAP of cross YOLO v3 reaches the highest value, 91.90%. When other conditions remain the same, cross PANet increases mAP by 0.95% compared with PANet, and by 2.76% compared to FPN. Compared to the improvement in accuracy, the inference time of cross YOLO v3 is 2 ms-higher than that of YOLO v3, which will not affect the real-time performance of target detection too much.

In Table 3, the influence of the combination of different anchor-setting methods and focal loss in SeaBuoys on cross YOLO v3 was experimentally compared. Introducing focal loss into the threshold method will reduce mAP by 2.77%, combining focal loss with the average method will increase mAP by 2.18%, and introducing focal loss into the select-all method will increase mAP by 2.71%, indicating that focal loss only plays a positive role on the basis of the average method and the select-all method.

Without the use of focal loss, the average method increases mAP by 4.29% compared with the threshold method, and the select-all method increases mAP by 2.86% compared to the threshold method. When focal loss is used, these two values are 9.24% and 8.34%. In the two indicators of TP and FP, cross YOLO v3 with the average method and focal loss also has the best performance. Different anchor-setting methods and focal loss will not bring any burden on the detection speed. The above demonstrates that the average method is the anchor-setting method with the best performance.

The mAP of cross YOLO v3, which has the best performance, is 0.52% higher than that of YOLO v4, but the inference time of cross YOLO v3 is 3 ms-higher than that of YOLO v4. In general, cross YOLO v3 meets the real-time requirements of sea-surface detection tasks.

### 4.3. Ablation Experiment in SeaShips

In order to verify the method proposed in this paper in a more comprehensive manner, the same ablation experiments were carried out in SeaShips, where 80% of the images were classified into a training set and the rest into a test set. First, the new baseline network cross YOLO v3 was tested. Second, ablation experiments were performed on different anchor-setting methods and focal loss under cross YOLO v3. The experimental results are displayed in the following table.

In Table 4, cross YOLO v3 achieved the highest mAP value in SeaShips, which was 78.44%. Compared with PANet, cross PANet increased the mAP by 0.64% and by 1.54% compared with FPN. The inference time of cross YOLO v3 was 2 ms-higher than that of YOLO v3, but it still had a good real-time performance.

In Table 5, the performance of the combination of different anchor-setting methods and focal loss in sea ships was experimentally compared. The results were similar to those in SeaBuoys. The introduction of focal loss into the threshold method reduced mAP by 1.67% while combining focal loss with the average method and the select-all method increased mAP by 5.83% and 4.89%, respectively.

Without using focal loss, the average method improves the mAP by 6.31% compared with the threshold method, and the select-all method increased mAP by 3.77% compared to the threshold method. When focal loss was used, these two values were 13.81% and 10.33%. Cross YOLO v3 with the average method and focal loss achieved the best performance on TP and FP indicators. The mAP of cross YOLO v3, which had the best performance, was 0.44% higher than that of YOLO v4, but the inference time of cross YOLO v3 was 3 ms higher than that of YOLO v4. For cross YOLO v3 itself, different optimization schemes in the experiment would not reduce the detection speed.

**Figure 5:**
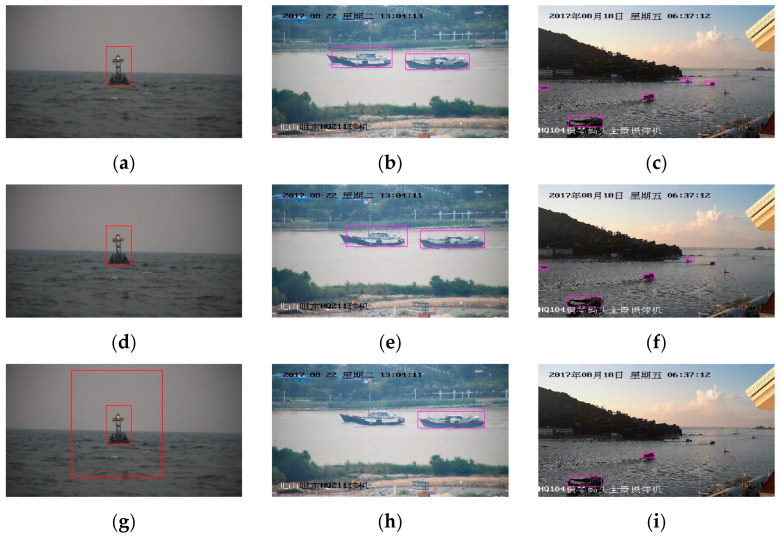
Part of the test results in the SeaShips and SeaBuoys datasets.

**Figure 6:**
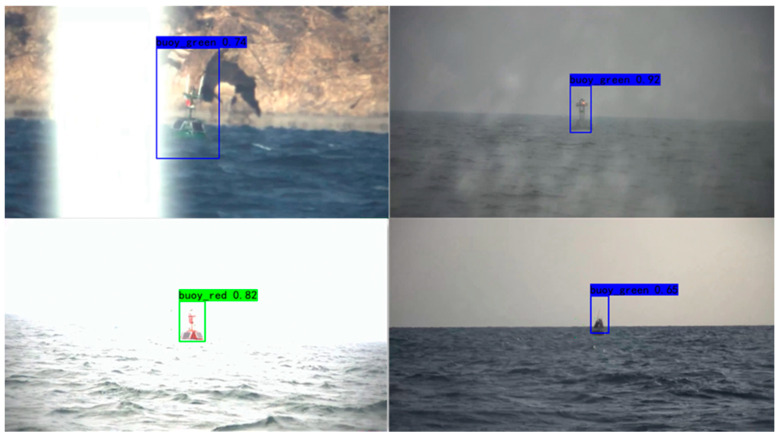
Part of the video detection results.

Figure 5 shows the detection results of cross YOLO v3 when different anchor-setting methods are used. Figure 5a–c shows the detection results of the average method, Figure 5d–f exhibit the detection results of the select-all method, and Figure 5g–i displays the detection results of the threshold method. Figure 6 shows the video detection results of cross YOLO v3. The video was collected from real sea conditions. There were many complex external factors in the video, such as changes of light, the sway of the boat, and water splashes.

The test results of SeaBuoys (left) and SeaShips (middle, right) are shown in Figure 5. The detection results in both datasets show that the Average method is the best anchor setting method.

Figure 6 shows the detection results of the cross YOLO v3 under the interference of external factors. These interferences include excessive light (left), splashing water (upper right) and too far shooting distance (lower right).

## 5. Discussion

The averaged method and the select-all method not only take the degree of overlap between the anchor and the ground-truth box as the standard when positive anchors are set, but also distribute positive anchors evenly to each feature map, and make full use of the semantic information and location information of different feature maps. Although the select-all method distributes the positive anchors uniformly to each feature map, excessive positive anchors are selected, which increases the difficulty of network training, leading to poorer experimental results than those of the average method. Moreover, in the experiment, it was found that focal loss only plays a positive role based on the average method and the select-all method. Focal loss and different anchor-setting methods do not change the structure of the network, so the detection speed will not be reduced.

Feature fusion has become a key component of the target detection network, and the feature pyramid structure is the most commonly used structure for feature fusion since it can combine strong positioning information in low-level features with strong semantic information in high-level features. The cross PANet structure integrates the strongest positioning information and semantic information in the multiscale feature maps more directly, which improves the detection accuracy. Compared with the FPN structure, the cross PANet structure will reduce the detection speed, but the real-time performance of cross YOLO v3 is still excellent.

## 6. Conclusions

In this paper, YOLO v3 was improved and applied to the actual sea-surface target detection. GIOU loss was added to the loss function, and cross PANet was proposed to replace the FPN structure in YOLO v3. These improvements contributed to a stronger baseline cross YOLO v3. By changing the anchor-setting methods during training, the accuracy of cross YOLO v3 to detect sea-surface targets can be improved without increasing the inference time. Experiments in two different datasets, SeaBuoys and SeaShips, showed that in the actual background where the number of sea-surface targets is small, compared to the threshold method, the average method and the select-all method could not only increase the mAP value of cross YOLO v3 without increasing the inference time, but also can be combined with focal loss to further enhance the detection accuracy of cross YOLO v3. In addition, the experimental results in SeaBuoys and SeaShips demonstrated that the combination of the average method and focal loss achieves the best performance.

## Figures and Tables

**Table 1:** Number and size of images of each category in the SeaBuoys dataset.

Category of Buoys	Number of Images	Proportion	Size of Image
buoy_1	887	0.154	1024×576
buoy_2	1000	0.174	1024×576
buoy_green	882	0.153	1024×576
buoy_red	987	0.172	1024×576
buoy_blue	1022	0.178	1024×576
buoy_yellow	973	0.169	1024×576

**Table 2:** Experimental results of cross YOLO v3 in the SeaBuoys dataset.

Baseline	Method	mAP (%)	Inference Time (ms)
YOLO v3	Threshold method	88.24	25
	Threshold method + GIOU loss	89.14	25
	Threshold method + GIOU loss + PANet	90.05	27
	Threshold method + GIOU loss + cross PANet	91.90	27

**Table 3:** The results of ablation experiments on different anchor-setting methods in the SeaBuoys dataset.

Baseline	Method	mAP (%)	Inference Time (ms)	TP	FP	GT
Cross YOLO v3	Threshold method	91.90	27	1159	72	1188
	Threshold method + focal loss	89.13	27	1151	85	
	Average method	96.19	27	1174	36	
	Average method + focal loss	98.37	27	1185	25	
	Select-all method	94.76	27	1169	42	
	Select-all method + focal loss	97.47	27	1175	34	
YOLO v4		97.85	24	1184	27	

**Table 4:** Experimental results of cross YOLO v3 in the SeaShips dataset.

Baseline	Method	mAP (%)	Inference Time (ms)
YOLO v3	Threshold method	76.10	25
	Threshold method + GIOU loss	76.90	25
	Threshold method + GIOU loss + PANet	77.80	27
	Threshold method + GIOU loss + cross PANet	78.44	27

**Table 5:** Results of ablation experiments on different anchor-setting methods in the SeaShips dataset.

Baseline	Method	mAP (%)	Inference Time (ms)	TP	FP	GT
Cross YOLO v3	Threshold method	78.44	27	1648	181	1823
	Threshold method + focal loss	76.77	27	1631	192	
	Average method	84.57	27	1708	109	
	Average method + focal loss	90.58	27	1742	88	
	Select-all method	82.21	27	1695	116	
	Select-all method + focal loss	87.10	27	1720	98	
YOLO v4		90.14	24	1741	91	
